# Occurrence of the large ostracod, *Chlamydotheca unispinosa* (Baird, 1862), in temporary waters of Montserrat, Lesser Antilles

**DOI:** 10.3897/zookeys.748.22323

**Published:** 2018-04-04

**Authors:** Robert E. Schmidt, Nathaniel F. Shoobs, Erin R. McMullin

**Affiliations:** 1 Bard College at Simon’s Rock, 84 Alford Rd., Great Barrington, Massachusetts, 01230, USA; 2 Academy of Natural Sciences of Drexel University, Department of Malacology, 1900 Ben Franklin Parkway, Philadelphia, Pennsylvania, 19103, USA; 3 Department of Biodiversity, Earth, and Environmental Science, Drexel University, 3201 Arch Street, Suite 240,​ Philadelphia. Pennsylvania, 19104, USA​

**Keywords:** COI, Cyprididae, West Indies, Podocopida

## Abstract

Four populations of the large freshwater ostracod, *Chlamydotheca unispinosa* (Baird, 1862), were discovered on the Caribbean island of Montserrat. These are the first records of the species on Montserrat and extend its known distribution approximately 113 km northwest and 63 km southeast of the closest known populations on Îles des Saintes (Guadeloupe) and Nevis, respectively. We provide the first DNA barcode for *C. unispinosa*, a 686 bp fragment of the COI gene which may be used for future comparative studies of this widely distributed species.

## Introduction

**Figure 1.**
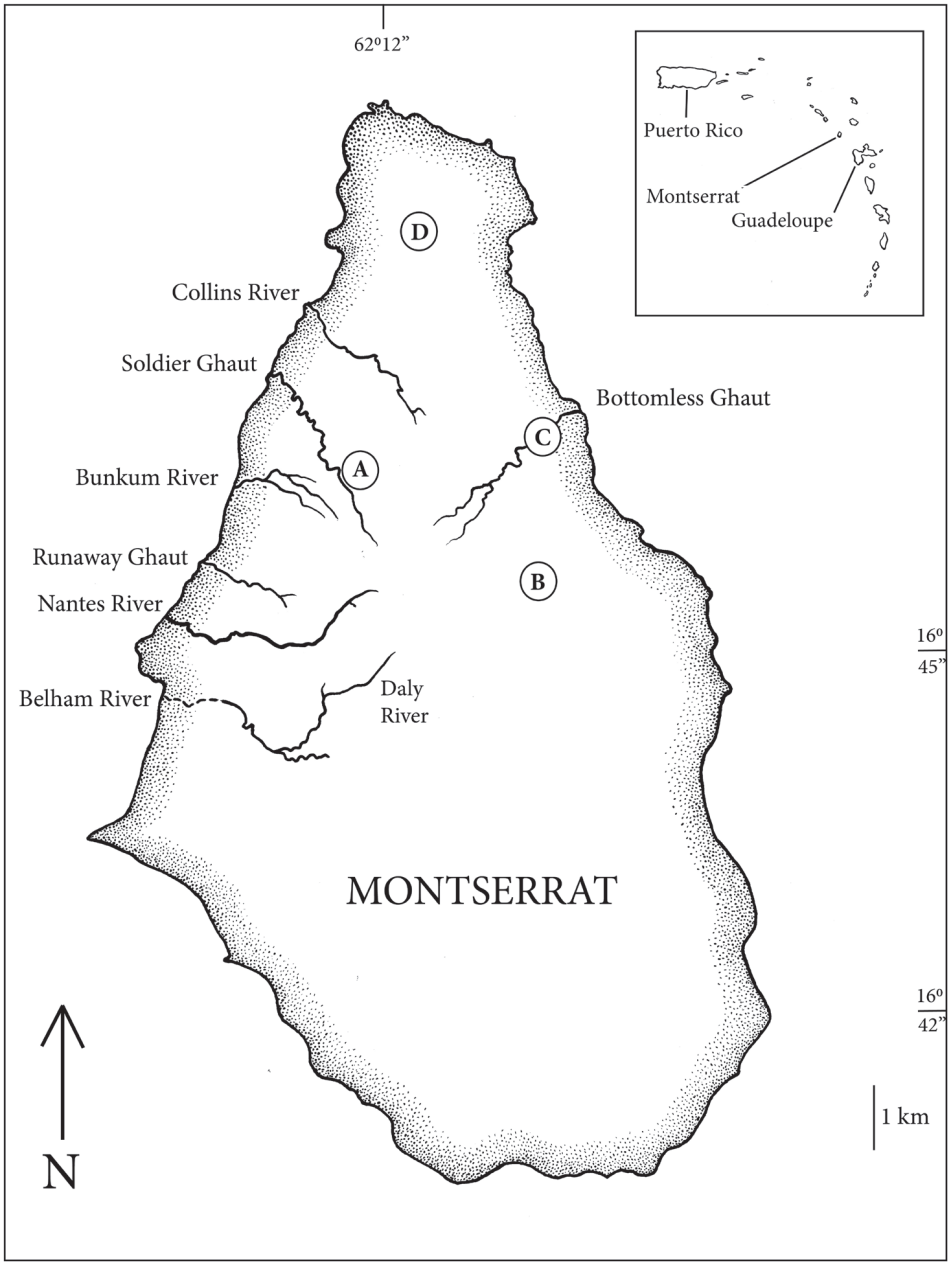
Map of Montserrat showing approximate collection localities of *Chlamydotheca unispinosa*. Empty valves were collected from a dry pool along Blackwood Allen Trail (**A**) and in a muddy puddle along Jack Boy Hill Trail (**B**). Living specimens were collected from Bottomless Ghaut (**C**) and Dowdye Pond (**D**).

The New World genus *Chlamydotheca* Saussure, 1858 contains primarily tropical large freshwater ostracods. There are 36 species (Martens and Savatenalinton 2011) with the majority from continental waters. Two species are recorded from Caribbean Islands, *C. barbadensis* Sharpe, 1910 and *C. unispinosa* (Baird, 1862). *Chlamydotheca barbadensis* was described from Barbados, recorded from northern South America and several islands off the South American coast (Broodbakker 1984), and with a disjunct distribution in Antigua, Barbuda, St. Eustatius, and St. Martin in the northern Lesser Antilles (Broodbakker 1984). *C. unispinosa* was described from Jamaica (Baird 1862); recorded from the Greater Antilles and the Bahamas; Nevis (Bass 2006), Marie Galante, and Îles des Saintes (Broodbakker 1984) in the Lesser Antilles; and Illinois (Evenson 1942), Maryland (Tressler 1947), Yucatan (Furtos 1936), Colombia (Roessler 1986), and south to Brazil (Tressler 1949). It has also been reported from Hawaii (Baird 1862; Eldridge and Miller 1997). Montserrat is a small volcanic island in the northern end of the Lesser Antilles (Figure 1). Volcanic eruptions and subsequent lahar flows from 1995–2012 destroyed a substantial portion of the freshwater lentic and lotic environments on the island (Barclay et al. 2007) and perhaps caused local extinctions of some aquatic organisms. Surveys of the remaining freshwater habitats led to this note reporting the presence of *C. unispinosa* in temporary epigean fresh waters of Montserrat, Lesser Antilles.

## Materials and methods

**Figure 2.**
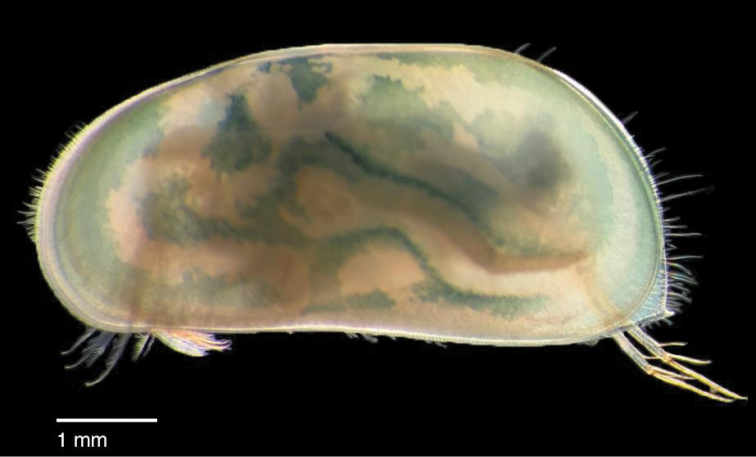
Preserved specimen of *Chlamydotheca unispinosa* from Bottomless Ghaut, Montserrat, approximately 4.1 mm long. Living individuals were colored bluish-green.

Living specimens of large ostracods were collected from shallow fresh water with fine mesh dip nets and fixed in 95 % ethanol. Empty valves located in dried temporary pools were collected by hand and stored dry. Three preserved specimens were deposited in the Academy of Natural Sciences, Philadelphia, two in 80% ethanol and one (DNA voucher) in 95 % ethanol (ANSP GI-19490). Empty valves were collected from a dry temporary pool dominated by the aroid, *Dieffenbachia seguine* (Jacq.) Schott, along the Blackwood Allen trail, Baker Hill, Montserrat (Fig. 1) on January 2, 2015 and January 10, 2017 (approximately 16°46'25.04"N, 62°12'27.17"W). Living specimens (Fig. 2) were collected from a shallow pool adjacent to the upstream edge of the road crossing over Bottomless Ghaut, Blake’s Estate, Montserrat on January 10, 2016 (16°46'45"N, 62°10'32"W). On January 17, 2017, empty valves were collected from a muddy temporary puddle along the Jack Boy Hill trail (approximately 16°45’46"N, 62°10’46"W) Trant’s Estate, Montserrat. Living specimens were collected from Dowdye Pond dominated by water lettuce, *Pistia stratiotes* L., along the road north of Gerald’s, St. Peter, Montserrat on January 16, 2018 (approximately 16°48’19.60”N, 62°11’35.78”W). The ostracods collected were identified as *C. unispinosa* (Baird 1862) by comparing our specimens with descriptions and illustrations in Roessler (1986). Additionally, illustrations of *C. barbadensis* show valves of a different shape from our specimens and without a point on the posterolateral margin (Sharpe 1910).

Genomic DNA was extracted from one entire animal using a DNeasy Blood & Tissue Kit (QIAGEN) and a 710bp region of the mitochondrial COI gene was amplified using HCO2198 and LCO1490 (Folmer et al. 1994). PCR products were purified using a Qiaquick PCR Purification Kit (QIAGEN) and sequenced using the same primers as the PCR (DNA Analysis Facility on Science Hill, Yale University). Complimentary forward and reverse sequences were aligned and edited in BioEdit (Hall 1999) and the resulting sequence was used in a BLAST search of the GenBank nucleotide database (blastn). A selection of DNA sequences similar to the Montserrat ostracod were downloaded and aligned using ClustalW (MEGA, Tamura et al. 2013). Alignments were edited and poorly aligning flanking regions were removed. Aligned sequences were translated, using an invertebrate mitochondrial genetic code table, into amino acid sequences to check for alignment errors. A neighbor-joining tree (bootstrap, 1000 replications) was constructed of COI sequences representing the Montserrat ostracod and the four most similar species published in GenBank, as well as a sequence from one more distantly related ostracod, *Conchoecetta cuminata* Claus, 1890 (Podocopida, Cytherideidae) as an outgroup (MEGA). Pairwise distances (p-distance, complete deletion) were calculated between the nucleotide sequences of the Montserrat ostracod and the four most similar published sequences, as well as one with the outgroup sequence of *C. cuminata* (MEGA).

## Results

**Figure 3.**
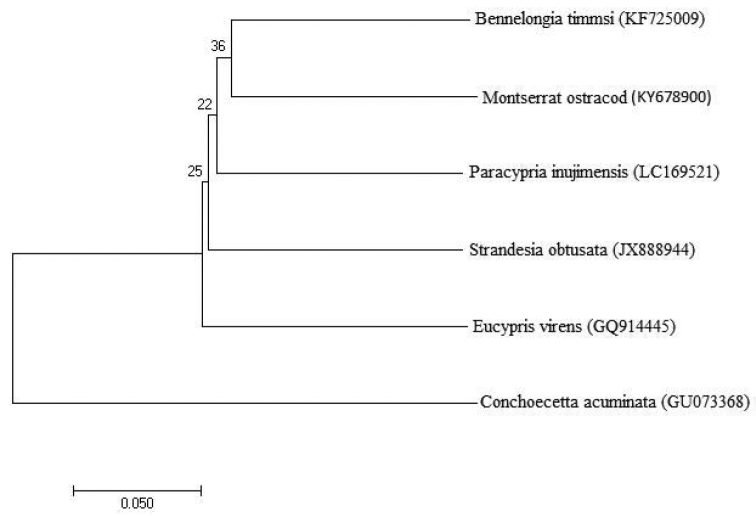
Neighbor-Joining tree of COI nucleotide sequences (codon positions 1, 2, and 3) from the Montserrat specimen of *Chlamydotheca unispinosa*, four of the most similar sequences and one outgroup. All positions containing gaps and missing data were eliminated. There was a total of 620 positions in the final dataset. Bootstrap values (1000 replicates) are shown at each node. Branch lengths represent evolutionary distances (p-distance) and are in the units of the number of base differences per site. All analyses were completed in MEGA 7 (Kumar et al. 2016).

The COI sequence generated for this Montserrat ostracod was deposited in GenBank (accession number KY678900). No COI, DNA, or amino acid sequence in GenBank was highly similar to the sequence obtained from the Montserrat ostracod. The most similar nucleotide and amino acid sequences included representatives from the genera *Bennelongia* De Decker & McKenzie, 1931; *Strandesia* Stuhlmann, 1888; *Eucypris* Vavra, 1891, and *Paracypria* Sars, 1910 (Fig. 3). The most similar DNA sequences had p-distances ranging from about 0.19 (*Bennelongia timmsi* Martens, Halse & Schön, 2013; #KF725009) to 0.22 (*Strandesia velhoi* Higuti, Schön, Audenaert, & Martens, 2013; #JX888939). The translated amino acid COI sequence of the Montserrat ostracod differed from its closest match, *S. velhoi*, by a p-distance of 0.02, and from *B. timmsi* by a p-distance of 0.04.

## Discussion

The nearest populations of *C. unispinosa* are on Nevis (Bass 2006) and Îles des Saintes (Broodbakker 1984), 113 km northwest and 63 km southeast of Montserrat, respectively. However, nearby islands to the northeast and northwest of Montserrat are inhabited by *C. barbadensis* (Broodbakker 1984).

Members of the genus *Chlamydotheca* can be found in lotic and lentic, permanent and temporary waters (Diaz and Lopretto 2011). The Montserrat specimens were all collected from seasonally dry locations; three temporary pools and a stream, Bottomless Ghaut, which is usually dry during the dry season. Substrate in the three temporary pools was muddy. Substrate in Bottomless Ghaut was gravel and cobble. Specimens collected were all large adults. Populations in Bottomless Ghaut and the puddle along Jack Boy Hill trail were small but there were probably thousands of empty valves in the dried pool along the Blackwood Allen trail and thousands of live animals in Dowdye Pond.

*Chlamydotheca unispinosa* belongs to the “*C. iheringi* group” (Roessler 1986). The center of diversity of this group of ostracods is in northern South America. *Chlamydotheca unispinosa* has the widest distribution of any member of the species group which may indicate that some or all of the populations outside of the South American continent are introduced. The records from Illinois, Maryland, and Hawaii are particularly suspect because the first two locations are temperate and all are vastly distant from northern South America. Some ostracods living in temporary freshwaters have desiccation-resistant eggs that can remain viable for >10 years (Boulton and Lloyd 1992) and could be transported great distances by wind, animals, or humans. Few studies have been done on this phenomenon in ostracods (Radzikowski 2013).

It is also possible that *C. unispinosa* is composed of several cryptic species. Studies comparing DNA sequences throughout the range of this species might determine whether cryptic species exist (Lara et al. 2010) or whether this species is particularly vagile. Unfortunately, these data do not currently exist. The COI sequence from this study (GenBank #KY678900) could be used to identify and compare similar sequences from other populations of *C. unispinosa*, particularly those reported from temperate regions.
